# Measurement of migration of a humeral head resurfacing prosthesis using radiostereometry without implant marking: An experimental study

**DOI:** 10.3109/17453674.2011.566133

**Published:** 2011-04-05

**Authors:** Olof Sköldenberg, Magnus Ödquist

**Affiliations:** Karolinska Institutet, Department of Clinical Sciences at Danderyd Hospital, Stockholm, Sweden.

## Abstract

**Introduction:**

Standard radiostereometric analysis of prosthetic migration requires that tantalum beads are inserted into the implant. For manufacturing reasons, this is not possible for humeral head resurfacing implants. We therefore used marker-free radiostereometry, developed for metal-backed acetabular cups, on a dummy model to validate the method for a humeral head resurfacing prosthesis.

**Material and methods:**

3 hemispherical resurfacing prostheses of different sizes were marked with tantalum beads and mounted in a sawbone. Standard and marker-free radiostereometry was then done repeatedly with gradual shifts of position of the prosthesis between each analysis. The marker-free algorithm was then compared to the standard to determine the accuracy.

**Results:**

The accuracy for marker-free radiostereometry was 0.22–0.47 mm for translations and 0.92–1.56 degrees for rotations.

**Interpretation:**

Based on our results, marker-free radiostereometry can be used to measure migration of humeral head resurfacing prostheses. This indicates that implant marking is not required when doing radiostereometry on humeral head resurfacing in clinical trials.

Radiostereometric analysis (RSA) (Selvik 1989) is the standard for measuring micromotion of orthopedic implants. With RSA, it is possible to get highly accurate three-dimensional (3-D) measurements from calibrated stereo radiographs. By making measurements over time, implant migration can be quantified and loosening predicted with high sensitivity (Kärrholm et al. 1994, Ryd et al. 1995). The method requires the insertion of tantalum markers into the skeleton and the implant to create 2 rigid bodies, called segments. The migration of the implant segment in relation to the skeleton segment for translation and rotation around the x-, y-, and z-axes (the 6 degrees of freedom) is then calculated.

Marking of an implant requires modification of the implant design. This is costly, and can potentially adversely affect the performance of the implant being studied. In many countries, the marking of implants is therefore prohibited by the regulatory authorities. The high density of the implant metal can also obscure the tantalum markers, resulting in loss of migration data (Kaptein et al. 2005).

For hemispherical metal-backed acetabular cups, an implant marker-free RSA algorithm (marker-less RSA) has been developed to address these problems. It has been found to be accurate in assessing penetration of femoral heads in hip arthroplasty and has also been used to determine acetabular cup migration (Valstar et al. 1997, Börlin et al. 2006, Zhou et al. 2006). To our knowledge, the method has not been used previously for migration analysis of a resurfacing prosthesis.

We determined the accuracy of marker-less RSA when used on a humeral head resurfacing prosthesis.

## Material and methods

### Experimental set-up

**Figure 1.**
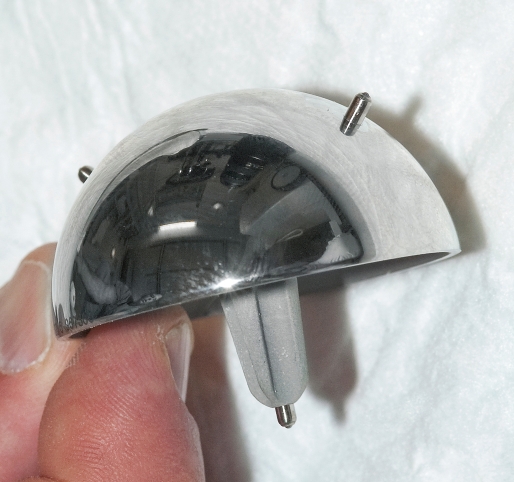
The Copeland humeral resurfacing head used in the study. The implant has been modified for standard RSA with 3 tantalum markers (arrows).

We used the Copeland humeral resurfacing head prosthesis (Levy et al. 2001) (Biomet, Warzaw, IN) in 3 sizes (3, 5, and 6). The manufacturer had marked each prosthesis with 3 tantalum markers at the outer periphery and the distal tip of the implant (Figure 1). The prostheses were implanted in a humeral phantom (Sawbones; Sawbones Europe, Malmö, Sweden) and in addition, 6 tantalum markers (1.0 mm) were placed in the sawbone to serve as the reference segment for the RSA analysis. The phantom was then placed above a uniplanar calibration cage (Uniplanar digital 43; RSA Biomedical AB, Umeå, Sweden). Digital radiographs (Bucky Diagnostic; Philips, Eindhoven, the Netherlands) were then taken using 1 fixed and 1 mobile X-ray source. The exposure was set to 125 kV and 2.5 mAs. The radiographs were saved in a standard dicom file format (resolution 254 dpi) and uploaded to a workstation. UmRSA 6.0 computer software (RSA Biomedical AB) was used for all measurements and migration analyses.

We performed the following procedure to measure the migration of the implant in relation to the sawbone:

1. The phantom was placed above the calibration cage at the point of intersection of the central X-rays.
2. One set of radiographs were taken (position 1, series 1).
3. The calibration cage, the X-ray tubes, and the phantom were repositioned.
4. One set of radiographs were taken (position 1, series 2).
5. The prosthesis was tilted and rotated by 0.5–1.0 degrees in relation to the sawbone to simulate migration of the implant.

Steps 1 to 5 were then repeated 5 times, giving us position 2, series 1 and 2, position 3, series 1 and 2, and so on. The markers in the sawbone formed the 3-D reference segment and were not altered between exposures.

### Standard RSA

**Figure 2.**
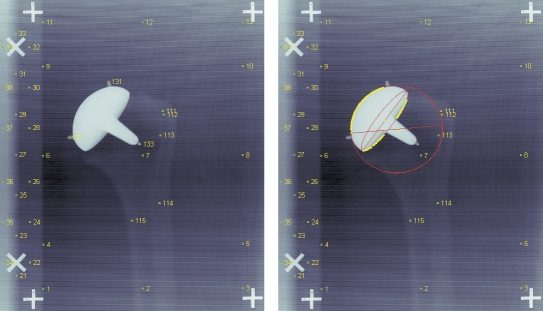
RSA analysis using standard and marker-less RSA. A. Standard RSA with 3 tantalum markers. B. Marker-less RSA using cup algorithm.

For standard RSA, the 3 tantalum markers on the prosthesis were first measured to obtain the prosthesis segment (Figure 2).

### Marker-less RSA

**Figure 3.**
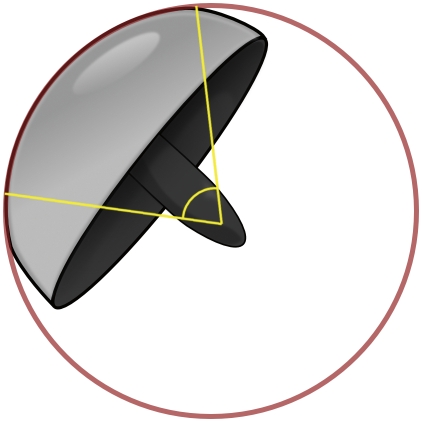
A schematic Copeland prosthesis showing the outer hemisphere and the inward slope of the rim. The red line describes a perfect circle. The yellow sector shows the 75 degrees on the hemisphere where points for marker-less RSA are placed.

The Copeland prosthesis is a hemisphere at its outer periphery, but the sides are tapered and slope inward towards the opening of the circle. We used a goniometer to place points on 75 degrees of the contour of the hemisphere (Figure 3) and then placed points on the opening circle of the prosthesis according to the method of the software. The software then automatically detected the boundaries of the prosthesis and calculated a prosthesis segment (Figure 2). The marker-less algorithm corresponds to a generalized hemisphere. The hemisphere's opening circle does not have to occur at the “equator” or have the same radius as the outer shell. The algorithm creates a prosthesis segment by adding points to the top of the hemisphere (“north pole”), the bottom of the hemisphere (“south pole”), the most anterior and posterior point of the opening circle, and the center of the hemisphere (Börlin et al. 2006).

### Precision

Precision—also called reproducibility—is the degree to which repeated measurements under unchanged conditions show the same results, and it refers to random errors. To calculate the precision of both RSA methods, the double measurements (series 1 and 2) taken at each of the 6 positions were analyzed for migration. The difference between the double measurements was then calculated, for example for x-translation (*xt*): *d_prec(xt)_ = xt_p1:1_ – xt_p1:2_* where *d_prec(xt)_* is the difference between position 1 series 1 (*p_1:1_*) and position 1 series 2 (*p_1:2_*). Since no migration of the implant in relation to the sawbone occurred, this difference represents the precision of the methods. Having used 3 different sizes of the prosthesis, we therefore had 18 double sets of radiographs on which to calculate precision.

### Accuracy

Accuracy is defined as the closeness of a true value (in this study, by standard RSA) to the most probable value, which has been derived from a series of measurements (in this study, by marker-less RSA). Accuracy includes both random and systematic errors. The accuracy of standard RSA was assumed to be perfect; i.e., standard RSA measures the true migration of the implant (Ostgaard et al. 1997, Valstar et al. 2000).

In order to calculate the accuracy of the marker-less RSA, the migration between 2 phantom positions was measured with both standard RSA and marker-less RSA. For instance, for y-translation (*yt*): *RSA_yt1–2_ = yt_p1_ – yt_p2_* where *RSA_yt1–2_* is the migration in y-translation of the prosthesis between position 1 (*p1*) and position 2 (*p2*) as measured with standard RSA. *ML_yt1–2_* is the same migration measured with marker-less RSA. The difference between these measurements was then calculated for y-translation (*yt*): *d_accur(yt)_ = RSA_yt1–2_ – ML_yt1–2_*. Ideally, this would be zero since both methods measured the same migration. To generate independent measurements, this was calculated pairwise for positions 1–2, 3–4, and 5–6. As 3 different sizes of the prosthesis were measured, we had 9 different sets of migration analysis performed to determine accuracy.

### Statistics

We defined the precision for standard and marker-less RSA as 2.11 SD (17 degrees of freedom) of the difference between the double examinations (*d_prec_*). We defined the accuracy for marker-less RSA as 2.26 RMS (9 degrees of freedom) of *d_accur_* (“root mean square”, a measure of the magnitude of varying quantity, since the difference between the two methods could be both positive and negative). We used SPSS statistical software version 17.0 for Windows.

## Results

The precision was good for translations when either of the RSA methods was used. For rotations, the precision was better for standard RSA (0.05– 0.33° as compared to 0.62–1.73° for marker-less RSA) (Table 1). The accuracy of marker-less RSA was 0.47 mm, 0.39 mm, and 0.22 mm for x-, y-, and z-translation. The accuracy was 1.56°, 1.10°, and 0.92° for x-, y-, and z-rotation (Table 2).

**Table 1.** Precision of standard and marker-less RSA

	Standard	Marker-less
	RSA	RSA
Translation (mm)		
x	0.03	0.22
y	0.02	0.15
z	0.11	0.16
Rotation (°)		
x	0.17	1.73
y	0.33	1.72
z	0.05	0.62

**Table 2.** Accuracy of marker-less RSA

	Accuracy
Translation (mm)	
x	0.47
y	0.39
z	0.22
Rotation (°)	
x	1.56
y	1.10
z	0.92

## Discussion

Our aim was to validate the marker-less RSA algorithm developed for acetabular cups when applied to a humeral head resurfacing prosthesis. We found the accuracy to be good for translations, but slightly less so for rotation along the x-axis (flexion/extension) and y-axis (anteversion/retroversion). This has been well documented by the authors who first described the method (Börlin et al. 2006).

High-precision measurements of a humeral head resurfacing prosthesis using RSA have not been reported previously, but loosening evident on plain radiographs has been described as subsidence or as increasing inclination angle of the implant (Rydholm et al. 1993, Levy et al. 2001). For marker-less RSA, this would correspond to y-translation and z-rotation of the implant.

There are advantages in using marker-free RSA systems when performing clinical trials involving hemispherical implants. Most importantly, the problem of marker occlusion is solved for prosthesis markers. However, bone markers are still necessary and they should be placed carefully to prevent occlusion by the implant. In standard RSA studies of acetabular cups, the loss of relevant patient data due to marker occlusion is typically 20–25% (Onsten et al. 1994, Thanner et al. 2000). In studies using marker-free systems, one can presumably expect a lesser amount of data loss. The marking of implants is costly, time-consuming, and can often—because of approval issues—only be done with the cooperation of the manufacturer. Marker-free software systems thus provide the possibility for industry-independent studies to be done at a lower cost.

Other marker-free RSA systems have been developed (Valstar et al. 1997, Kaptein et al. 2003). The system that seems to be most precise is Model-based RSA 3.0 (MbRSA) (Medis, Leiden, the Netherlands). MbRSA requires a 3-D surface model of the implant to estimate migration. The method is highly accurate when compared to either standard RSA or hemispherical cup algorithms (Baad-Hansen et al. 2007). One advantage of the method is that it can be used on all types of implants, not only implants of hemispherical design (Seehaus et al. 2009). The method, however, requires either reverse engineering of the implant with laser scanners or that the manufacturer supplies the researcher with CAD models of the implant. The method also assumes a perfect manufacturing process; any inaccuracies in the size and/or the surface of the prosthesis will reduce the precision.

3 reports have been published in which RSA has been used to study the migration of a resurfacing implant (Glyn-Jones et al. 2004, Itayem et al. 2005, 2007). All described migration of the Birmingham hip resurfacing prosthesis, and all studies used standard RSA with tantalum marker beads inserted in the prosthesis. This is understandable, since the marker-less method cannot be used for resurfacing implants when, as is the case for the Birmingham hip, a metal acetabular component obscures the resurfacing implant surface.

To our knowledge, there has only been one published clinical study where marker-less RSA was used. Zhou and co-workers (2006) performed a randomized controlled trial comparing migration of a porous-coated hemispherical acetabular cup with two types of articulation: ceramic-on-ceramic articulation and metal-on-polyethylene. They found no difference in cup migration between the 2 groups. They also compared the precision of standard and marker-less RSA, and their results regarding precision are similar to ours. They concluded that the use of marker-less RSA—although slightly less accurate than standard RSA, especially for x-and y-rotation—is possible in clinical trials. As in our study, they also found a good accuracy for translations.

Our study has some limitations. Firstly, no method with more accuracy than standard RSA was available with which to verify the difference between standard RSA and marker-less RSA. In phantom studies like this one, however, the accuracy of standard RSA is very close to perfect (Ostgaard et al. 1997, Valstar et al. 2000). As we only intended to describe the migration in a phantom model, we believe that for all practical purposes this assumption is correct. The good precision of standard RSA for all degrees of freedom in our study (Table 1) strengthens this hypothesis. Secondly, we had no access to cadaver humerus in which to implant the prosthesis. Thus, the effect of bone and soft tissue in reducing the precision could not be accounted for. As in all RSA studies, it is therefore important to perform double measurements when using marker-less RSA in clinical trials (Valstar et al. 2005).

In conclusion, marker-less RSA is a simple and accurate alternative to standard RSA to describe the migration of a humeral head resurfacing prosthesis. We plan to use the system in our future clinical trials.
